# Case Report: Lung Ultrasound for the Guidance of Adjunctive Therapies in Two Invasively Ventilated Patients with COVID-19

**DOI:** 10.4269/ajtmh.20-0538

**Published:** 2020-09-09

**Authors:** Charalampos Pierrakos, Rachid Attou, Enrica Iesu, Hugues Baelongandi, Patrick M. Honore, Lieuwe D. J. Bos, Marcus J. Schultz, David De Bels

**Affiliations:** 1 Department of Intensive Care, Brugmann University Hospital, Université Libre de Bruxelles, Brussels, Belgium;; 2 Department of Intensive Care, Amsterdam UMC, University of Amsterdam, Amsterdam, The Netherlands;; 3 Department of Anesthesiology, Brugmann University Hospital, Université Libre de Bruxelles, Brussels, Belgium;; 4 Laboratory of Experimental Intensive Care and Anesthesiology (LEICA), Amsterdam UMC, University of Amsterdam, Amsterdam, The Netherlands;; 5 Department of Respiratory Medicine, Amsterdam UMC, University of Amsterdam, Amsterdam, The Netherlands;; 6 Mahidol–Oxford Tropical Medicine Research Unit (MORU), Mahidol University, Bangkok, Thailand;; 7 Nuffield Department of Medicine, University of Oxford, Oxford, United Kingdom

## Abstract

Two patients with respiratory failure due to confirmed COVID-19 were examined using bedside lung ultrasound (LUS) shortly after intubation and start of invasive ventilation. In the first patient, LUS revealed extensive atelectatic areas. A recruitment maneuver was applied, resulting in some reaeration of areas that showed atelectasis, and some improvement in oxygenation was observed. Oxygenation improved further with the use of prone positioning. In the second patient, LUS showed diffuse abnormalities without atelectatic areas, and ventilation proceeded without a recruitment maneuver but with prone positioning. These two cases illustrate how LUS could be useful in identifying different lung morphologies early after the start of invasive ventilation and help decide on adjunctive therapies. This has possible implications for ventilator management in resource-limited settings, with limited availability of chest computed tomography and blood gas analyzers. Tailoring invasive ventilation based on LUS findings early after the start of invasive ventilation is feasible, but this should be further evaluated in future studies.

## INTRODUCTION

The world is increasingly struggling with the COVID-19 pandemic, causing tens of thousands of hospitalizations worldwide each week. In many low- and middle-income countries, these numbers are rising rapidly.^1^ An estimated 5% of hospitalized patients will need admission to an intensive care unit (ICU), mainly for receiving invasive ventilation,^2^ and a personalized ventilator approach may be needed.^3^ Chest computed tomography (CT) has been proposed to guide the assessment of reaeration, given that surrogate markers like global oxygenation and respiratory system compliance might be misleading.^4^ Lung ultrasound (LUS), a noninvasive imaging technique that can be used at the bedside, is an attractive alternative to chest CT,^5^ especially in settings where resources are restricted. Here, we present two cases of COVID-19 in which LUS contributed to the assessment of loss of aeration of lung tissue.

### The global LUS score for semi-quantification of lung aeration.

One frequently used tool to quantify extend of pulmonary pathologies is the so-called global LUS score, in which LUS patterns across 12 lung regions are caught in a numerical score.^5^ An “A-pattern” (i.e., repeating horizontal [A-] lines parallel to the pleural line, suggesting normal aeration) is scored “0”; a “B-pattern” (i.e., three or more vertical [B-] lines starting from the pleural line and reaching the bottom of the screen, suggesting partial loss of aeration) is scored “1” if lines are well spaced, and “2” if lines are confluent; and a “C-pattern” (i.e., consolidation, suggesting near-complete to complete loss of aeration) is scored “3.” The individual scores per region are summed into a global LUS aeration score, which thus ranges from “0” (normal aeration in all regions) to “36” (severe abnormal aeration in all regions).

## CASE REPORTS

**Figure 1.**
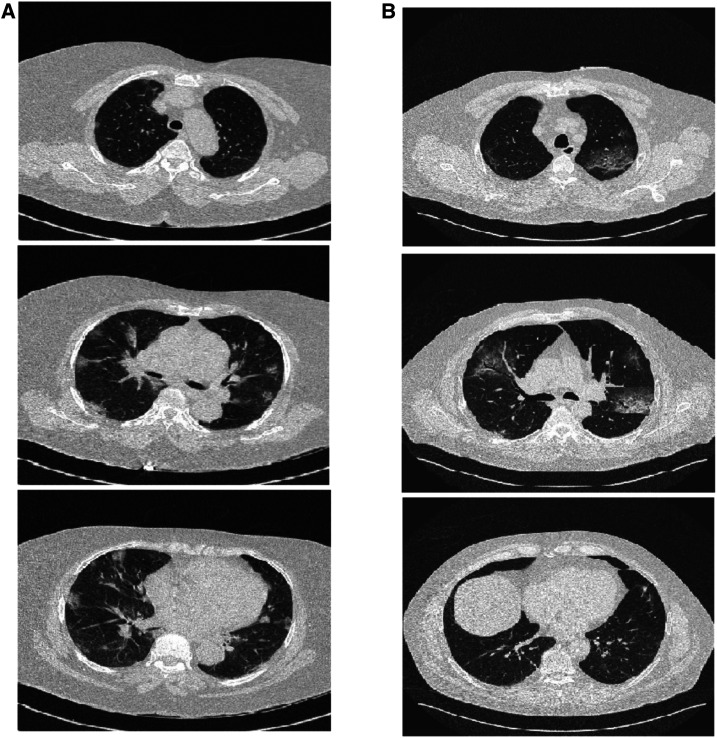
Chest computed tomography on the day of hospital admission for patients A (left) and B (right) at 10 and 12 days before intubation.

Patient A, a 66-year-old woman with a body mass index of 36 kg/m^2^, was intubated 10 days after hospitalization for reverse transcriptase-PCR (RT-PCR)–confirmed COVID-19, and 3 days after admission to the ICU. The chest CT obtained on the day of hospitalization showed typical ground-glass opacities with interlobular and intralobular septal thickening (“crazy paving”) involving 30% of the lungs (Figure 1; panel A). Two days before the start of invasive ventilation, the global LUS score was 15, with none of the examined regions scoring “3.”

**Figure 2.**
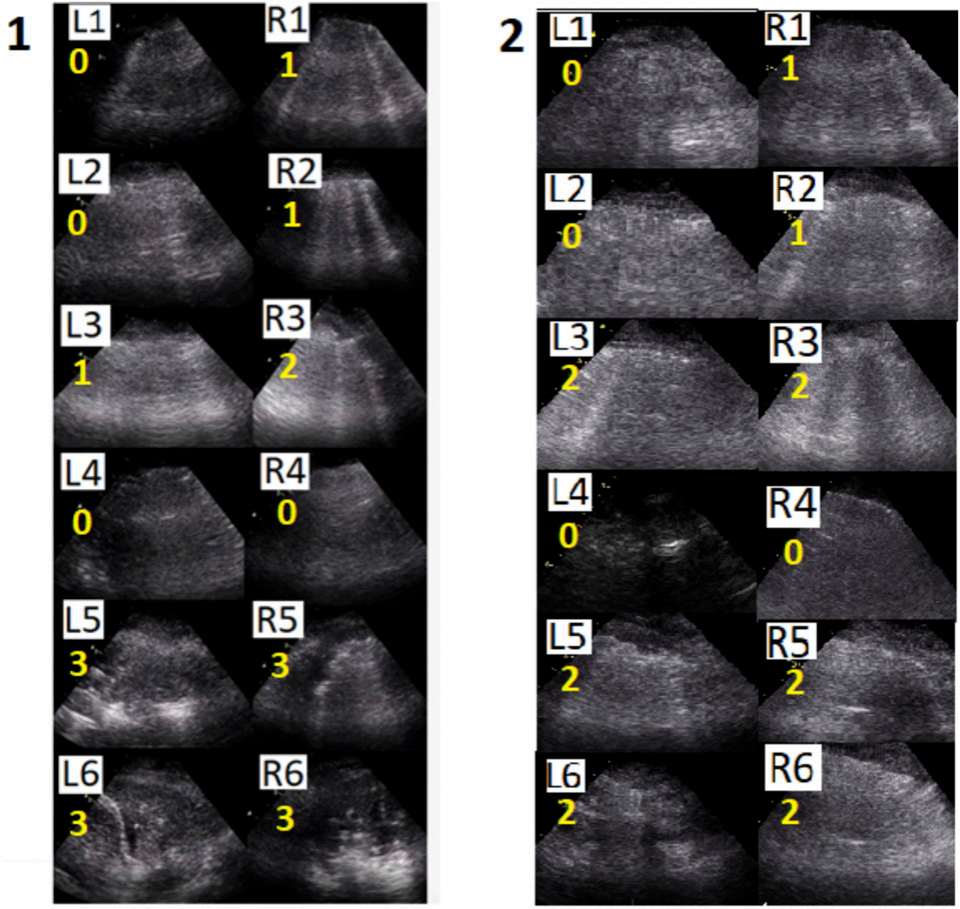
Lung ultrasound images for patient A, before (panel 1) and after the recruitment maneuver (panel 2). For each image, the corresponding score is presented (0–3). L = left hemithorax; R = right hemithorax; 1, 2 = anterior thoracic areas; 3, 4 = lateral thoracic areas; 5, 6 = posterior thoracic areas.

**Figure 3.**
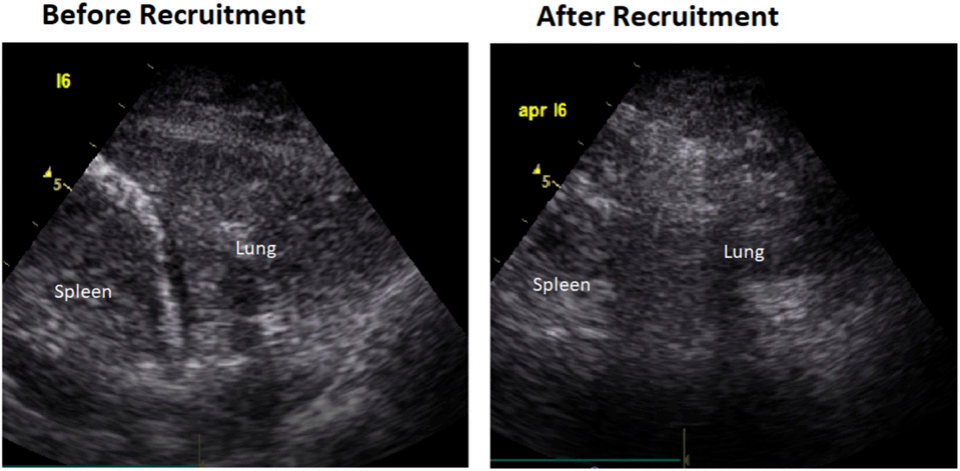
The effect of using recruitment maneuvers on the left posterior areas of the lung in patient A.

Invasive ventilation started with a tidal volume of 7 mL/kg predicted body weight, 10 cm H_2_O positive end-expiratory pressure (PEEP), and 100% FiO_2_. Oxygenation was poor, with a PaO_2_ to FiO_2_ ratio of 61 mmHg, and the pulmonary system appeared stiff, with a compliance of 24 mL/cm H_2_O at a driving pressure of 14 cm H_2_O. At that moment, the global LUS score was 17, with more regions scoring “3” at the dorsal site (Figure 2; panel 1). A significant recruitment maneuver, consisting of continuous airway pressure at 60 cm H_2_O for 40 seconds, resulted in some improvement in the global LUS score from 17 to 14, mainly through improvements in aeration of dorsal areas (Figure 2; panel 2 and Figure 3). An increase in oxygen saturation from 88% to 100% was observed, although need for high FiO_2_ remained. Prone positioning was applied, resulting in a sturdy improvement in the PaO_2_ to FiO_2_ ratio from 120 to 270 mmHg and an increase in the pulmonary system compliance from 24 to 30 mL/cm H_2_O at a driving pressure of 11 cm H_2_O.

Patient B, a 60-year-old man with a body mass index of 37 kg/m^2^, was intubated 12 days after hospitalization for RT-PCR–confirmed COVID-19, and 6 days after ICU admission. A chest CT performed on the day of hospitalization showed typical ground-glass and crazy paving involving 30% of the lungs (Figure 1, panel B). One day before intubation, the global LUS score was 11, with none of the examined areas scoring “3.”

**Figure 4.**
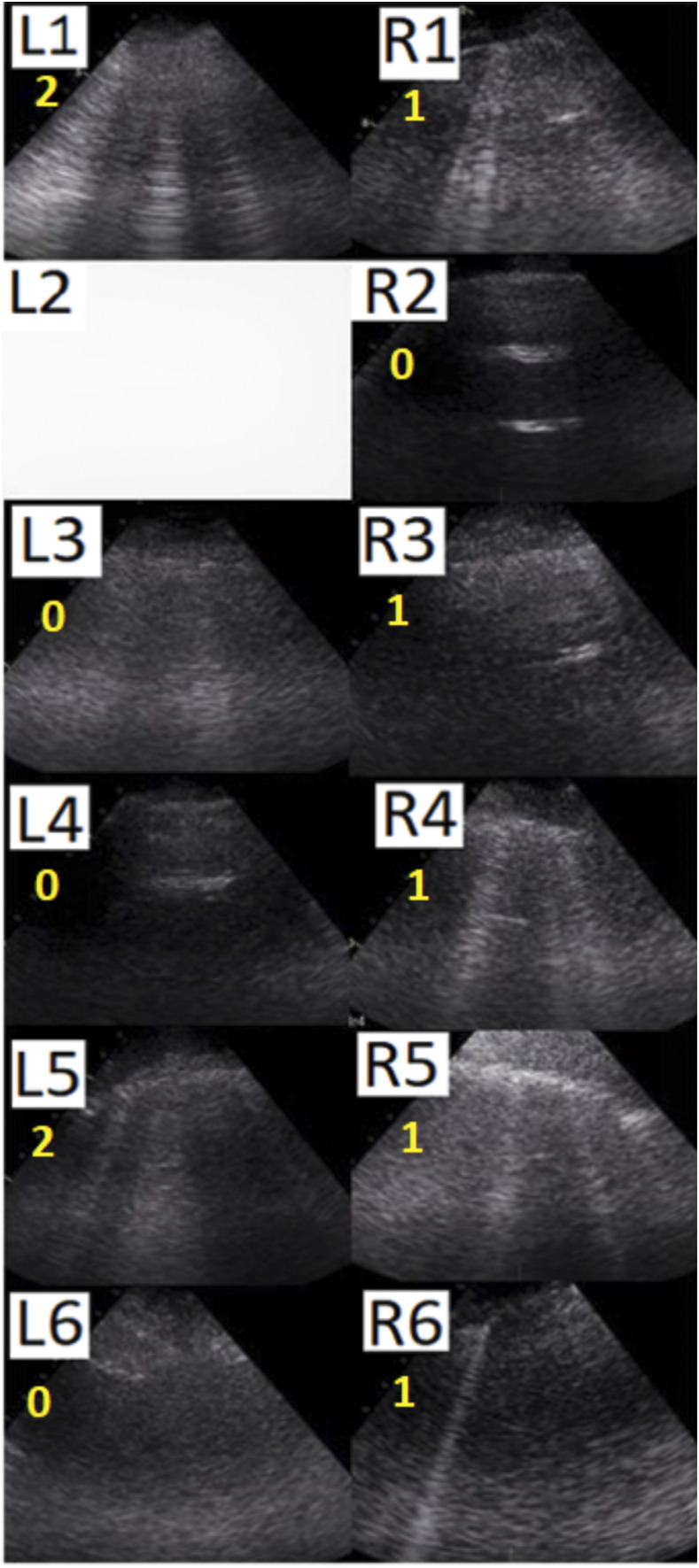
Lung ultrasound images for patient B. L = left hemithorax; R = right hemithorax; 1, 2 = anterior thoracic areas; 3, 4 = lateral thoracic areas; 5, 6 = posterior thoracic areas.

Invasive ventilation started with a tidal volume of 6 mL/kg predicted body weight, 10 cm H_2_O PEEP, and 100% oxygen. Oxygenation was poor with a PaO_2_ to FiO_2_ ratio of 116 mmHg, and the respiratory system appeared stiff with a compliance of 40 mL/cm H_2_O at a driving pressure of 11 cm H_2_O. At the start of invasive ventilation, the global LUS score was 9, with no single region scoring “3” (Figure 4). Ventilator settings were not changed, recruitment maneuvers were not performed, and the patient was placed in the prone position. After 16 hours of ventilation with 15 cm H_2_O PEEP in the prone position, oxygenation had improved, with a rise in the PaO_2_ to FiO_2_ ratio from 116 to 150 mmHg. Pulmonary system compliance had not changed, 37 mL/cm H_2_O at a driving pressure was of 13 cm H_2_O.

## DISCUSSION

These two case reports of COVID-19 illustrate the following: 1) LUS can be used to identify the lung morphological pattern of loss of aeration at the start of invasive ventilation, 2) differences in LUS patterns observed between these two patients included extent (i.e., a difference in the global LUS aeration score) and location (in patient A mainly located at the dorsal side) of loss of aeration, and 3) LUS could help in the decision to apply adjunctive therapies, especially the latter finding may have meaning for low-resource settings.

Several reports have shown the usefulness of LUS as a diagnostic tool in COVID-19.^6–9^ The findings in the cases described here point to the possibility to use LUS as a prediction tool for the effectiveness of adjunctive therapies. The patient with extensive atelectasis at the dorsal site responded poorly to a significant recruitment maneuver but responded well to prone positioning. The patient with less-extensive abnormalities also responded well to prone positioning. These findings are in accordance with those in a previous study that suggest that possibly patients with a focal morphology characterized by lobar, posterior atelectasis are more likely to be benefitted from prone position than from recruitment maneuvers.^10^

In these two cases, a recruitment maneuver and prone positioning were used as “rescue” therapies for refractory and life-threatening hypoxemia. Improvement in oxygenation, however, should never be considered as goal in itself, as improvement in oxygenation may not associated with a better outcome.^11,12^

Although in patient A oxygenation improved after the recruitment maneuver, only a minimal improvement in the global lung aeration was seen. Seen the fact that lung compliance remained low, hyperinflation may have occurred, which cannot be detected with LUS.^13,14^ Furthermore, regions with a “C-pattern” were located exclusively on the dorsal site that is not easily recruitable. COVID-19 patients with extensive consolidations in dorsal lung parts may thus respond better to prone positioning than to recruitment maneuvers. It should be noted that patient B did not receive a recruitment maneuver, and we can only conclude that prone positioning leads to a satisfying improvement in oxygenation.

Lung ultrasound is attractive because ultrasound machines are widely available, mostly portably, and thereby the technique can be used even in resource-limited settings and eliminates the need for transport to the radiology department, for example, for CT scanning.^15,16^ Furthermore, LUS can be repeated many times as it is a bedside, radiation-free imaging technique. Of importance, repetition of lung imaging might be relevant in the context of COVID-19 as the morphology of the lung may change over time. Last but not least, this advantage permits the assessment of lung aeration immediately after any change in ventilator parameters. Although it would have been attractive to perform follow-up LUS in the prone position, it is impossible to score and, thus, compare the chest regions that are scored in the supine position.

It has been speculated that poor lung compliance in the context of COVID-19 could indicate a higher likelihood of extensive atelectasis and, possibly, positive response to recruitment maneuvers.^17^ Nevertheless, this hypothesis has not been confirmed by clinical data where it has been shown that lung compliance is unrelated to the extent of parenchymal involvement in patients with COVID-19.^18^ Both patients presented here had abnormal dynamic compliance of the respiratory system that did not improve to normal values despite clinical improvement. It is highly likely that our patient had decreased chest wall compliance because of severe obesity, which is a common comorbidity in severe COVID-19 pneumonia. This is further supportive of the simultaneous use of LUS and lung mechanics. A decrease in LUS aeration score without a concomitant improvement of compliance may imply overdistention of lung units, whereas slight changes in compliance without changes in the LUS score may imply position-related changes in chest wall compliance.

In conclusion, we report our experience with LUS in two COVID-19 patients with refractory hypoxia who required invasive ventilation and adjunctive therapies for refractory hypoxemia. The two presented cases suggest the need for a personalized approach in use of adjunctive therapies in patients with COVID-19. Lung ultrasound could be an attractive bedside tool to guide their use.
